# Development and validation of a fourteen- innate immunity-related gene pairs signature for predicting prognosis head and neck squamous cell carcinoma

**DOI:** 10.1186/s12885-020-07489-7

**Published:** 2020-10-20

**Authors:** Fujun Zhang, Yu Liu, Yixin Yang, Kai Yang

**Affiliations:** 1 Department of Oral and Maxillofacial Surgery, the First Affiliated Hospital of Chongqing Medical University, No 1. Youyi Road, Yuzhong District, Chongqing, 400016 China; 2 Department of Pharmacy, the First Affiliated Hospital of Chongqing Medical University, Chongqing, 400016 China

**Keywords:** TCGA, Bioinformatics, HNSCC, Immune-related gene pairs, Riskscore

## Abstract

**Background:**

Immune-related genes is closely related to the occurrence and prognosis of head and neck squamous cell carcinoma (HNSCC). At the same time, immune-related genes have great potential as prognostic markers in many types of cancer. The prognosis of HNSCC is still poor currently, and it may be effective to predict the clinical outcome of HNSCC by immunogenic analysis.

**Methods:**

RNASeq and clinical follow-up information were downloaded from The Cancer Genome Atlas (TCGA), the MINiML format GSE65858 chip expression data was downloaded from NCBI, and immune-related genes was downloaded from the InnateDB database. Immune-related genes in 519 HNSC patients were integrated from TCGA dataset. By using multivariate COX analysis and Lasso regression, robust immune-related gene pairs (IRGPs) that predict clinical outcomes of HNSCC were identified. Finally, a risk prognostic model related to immune gene pair was established and verified by clinical features, test sets and GEO external validation set.

**Results:**

A total of 699 IRGPs were significantly correlated with the prognosis of HNSCC patients. Fourteen robust IRGPs were finally obtained by Lasso regression and a prognostic risk prediction model was constructed. Risk score of each sample were calculated based on Risk models and divided into the high-risk group (Risk-H) and low Risk group (Risk-L). Risk models were able to stratify the risk in patients with TNM Stage, Age, gender, and smoking history, and the AUC > 0.65 in training set and test set, shows that 14-IRGPs signature in patients with HNSCC has excellent classification performance. In addition, 14-IRGPs had the highest average C index compared with the prognostic characteristics and T, N, and Age of the 3 previously reported HNSCC.

**Conclusion:**

This study constructed 14-IRGPs as a novel prognostic marker for predicting survival in HNSCC patients.

## Background

Human head and neck squamous cell carcinoma (HNSCC) is one of the most common tumors today, with approximately 550,000 people worldwide suffering from this disease each year, and approximately 300,000 patients die [1]. Long-term repeated inflammatory stimuli are considered to be one of the main causes of the disease, including smoking, drinking, repeated trauma, and human papillomavirus (HPV) infection [2]. HNSCC is characterized by high proliferative, regional lymph node metastasis and poor prognosis [3]. It is urgent to investigate the development of novel and sensitive HNSCC tumor prognostic markers to reduce the number of HNSCC patients not diagnosed prior to the onset of invasive disease.

Cancer immunotherapy aims to enhance the activity of the immune system to fight cancer, has always been the main driving force of personalized medicine [4, 5]. In recent decades, immunotherapy has developed rapidly and has become a treatment for many cancers [6]. The expression of PD-L1 is usually higher in HNSCC tumors with a positive rate of 46 to 100% in several studies [7]. Tadalafil and anti-tumor vaccine-mediated immune rejection reversal also lead to up-regulation of PDL1 in recurrent HNSCC, suggesting that immunological checkpoint treatment may be effective in patients with HNSCC [8]. In 2016, the US food and drug administration (FDA) approved the first immunotherapy treatments- nivolumab and pembrolizumab for patients with recurrent (HNSCC with platinum-based regimens that are difficult to treat) [9]. Although these findings support the importance of immunology in HNSCC, the molecular mechanisms remain unclear, especially for immune-related genomic effects. With the advent of public large-scale gene expression data sets, cancer researchers have been able to accurately identify tumor-related prognostic biomarkers [10]. Li et al analyzed the prognostic value of IRGPs to develop individualized immune features that improve prognosis in patients with non-squamous non-small cell lung cancer [11]. However, the clinical relevance and prognostic significance of IRGPs in HNSCC have not been studied in depth.

In this study, we integrated immune-related genes in 519 HNSCC patients based on the TCGA dataset. Multivariate COX analysis and Lasso regression were used to identify robust IRGPs that predicted HNSCC clinical outcomes and establish a risk prognosis model related to immune gene pairs. IRGPs was found to be a strong prognostic biomarker and predictor of HNSCC.

## Methods

### Data collection and processing

In April 30, 2019, RNA-seq data and the latest clinical follow-up information were downloaded from TCGA using GDC API, including 612 RNA-seq data samples. Similarly, a set of chip data set GSE65858 in MINiML format were downloaded from NCBI, including the expression profile data and clinical follow-up information of 270 HNSCC sample. All patients underwent surgery with a negative surgical margin, receive no adjuvant or neoadjuvant therapy. A total of 1039 immune-related genes (removing the name-repeated gene) were downloaded from the InnateDB database (https://www.innatedb.com/).

**Fig. 1:**
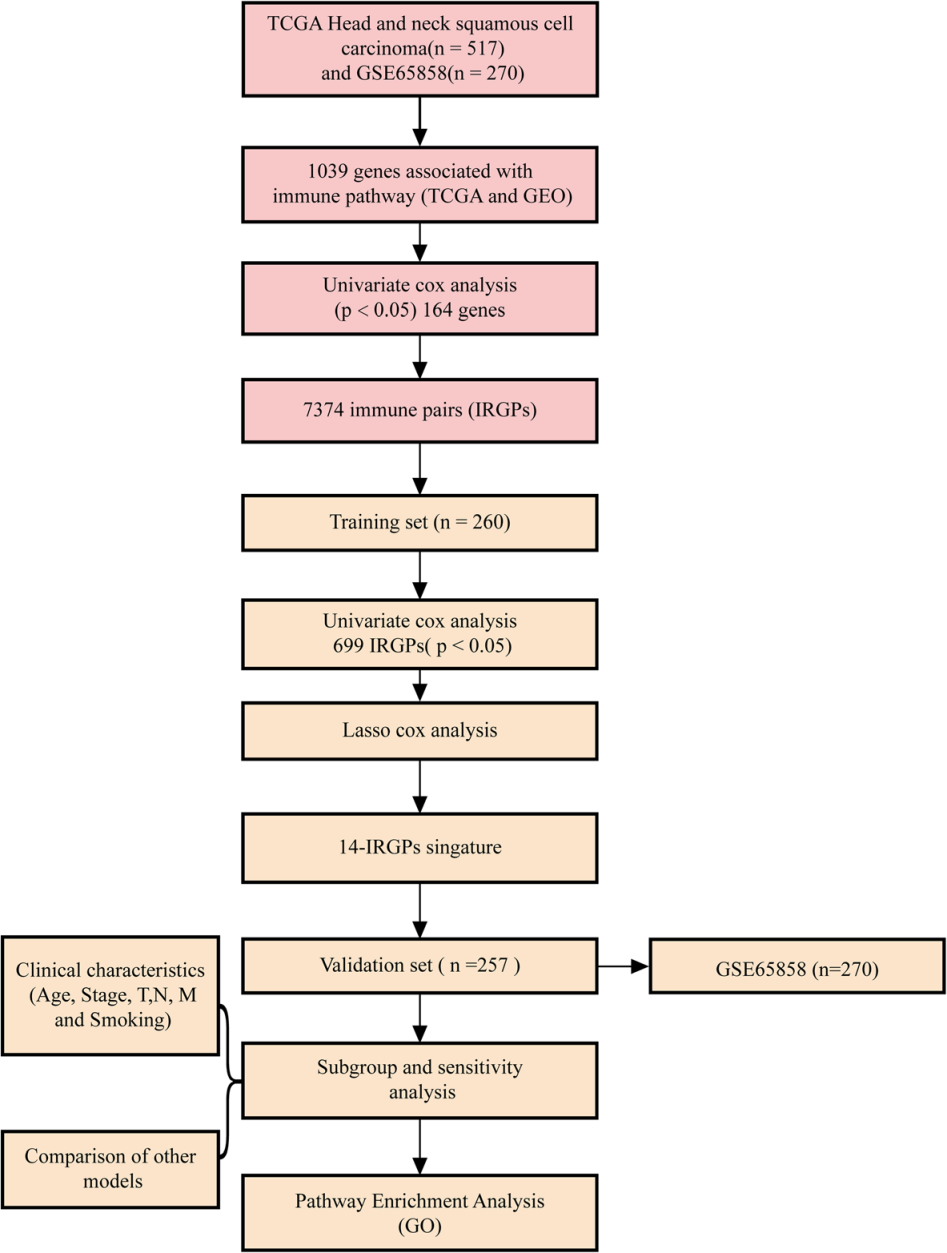
Work flow chart

For the TCGA RNAseq data, we screened 517 tumor samples with follow-up information and OS > 0, extracted the expression profile of the immune-related gene set and removed the gene with 0 expression level in 50% of the samples. For chip data sets, we screened samples with follow-up information and OS > 0, R package GEOquery was used to map the chip probes to GeneSymbol, the probes was mapped to multiple genes were removed, multiple probes were mapped to a single gene to take the median, gene expression profile were obtained, and the expression profile of the immune gene set were extracted. The clinical information of TCGA and GEO patients is shown in Table 1. The workflow is shown in Fig. 1.

**Table 1:** Clinical information of data sets

Characteristic		TCGA dataset (***n*** = 517)	GSE65858 (***n*** = 270)
**Age (years)**	<=60	256	41
	> 60	261	229
**Survival status**	Living	297	176
	Dead	220	94
**Gender**	female	136	47
	male	381	223
**Grade**	G 1	61	–
	G 2	303	--
	G 3	124	--
	G 4	7	--
**pathologic_T**	T 1	36	35
	T 2	149	80
	T 3	136	58
	T 4	184	97
**pathologic_N**	N 0	244	94
	N 1	83	32
	N 2	162	132
	N 3	9	12
**pathologic_M**	M 0	491	263
	M 1/ M X	23	7
**Tumor stage**	Stage I	27	18
	Stage II	81	37
	Stage III	93	37
	Stage IV	316	178
**Smoking**	Non-Smoking	117	48
	Smoking	388	222

### Sample grouping

For better model building and validation, we randomly divided the TCGA data set into two groups, one as a training set (*N* = 260), one as an internal validation set (*N* = 259), and the GSE65858 data set as an independent external validation set. During the random grouping process of TCGA, we kept the two groups of samples similar in age distribution, clinical stage, follow-up time, and proportion of patient deaths, while the number of samples after clustering the gene expression profiles of the two groups was close to each other, and the statistical characteristics of the two samples are shown in Table 2.

**Table 2:** Sample statistics for training set and validation set

Characteristic		TrainingSet (***n*** = 260)	TestingSet (***n*** = 257)	***p*** value
**Age (years)**	<=60	124	132	0.38
	> 60	136	125	
**Survival status**	Living	148	149	0.878
	Dead	112	108	
**Gender**	female	69	67	0.983
	male	191	190	
**Grade**	G 1	29	32	0.653
	G 2	152	151	
	G 3	65	59	
	G 4	5	2	
**pathologic_T**	T 1	19	17	0.886
	T 2	72	77	
	T 3	70	66	
	T 4	95	89	
**pathologic_N**	N 0	131	113	0.004
	N 1	50	33	
	N 2	64	98	
	N 3	6	3	
**pathologic_M**	M 0	244	247	0.1
	M 1/ M X	16	7	
**Tumor stage**	Stage I	16	11	0.444
	Stage II	40	41	
	Stage III	52	41	
	Stage IV	152	164	
**Smoking**	Non-Smoking	55	62	0.511
	Smoking	198	190	

### Construction of IRGPs

A total of 539,241 gene pairs were obtained by randomly permutation and combination of 1039 immune genes. For arbitrary gene i (*IRG*_*i*_) and gene j (*IRG*_*j*_), *IRGP*_*ij*_,were calculated. The IRG*P* values were defined as follows:

$$ {IRGP}_{ij}=\left\{\begin{array}{c}1,\kern0.75em I{RG}_i<I{RG}_j\\ {}0,\kern0.75em I{RG}_i\ge I{RG}_j\end{array}\right. $$

Where IRG indicates the amount of gene expression, we calculated all IRGPs values for all samples and further filtered IRGPs with a standard deviation of 0, a total of 18,182 IRGPs were obtained.

### Univariate cox survival analysis

Univariate Cox proportional hazard regression analysis was performed on each IRGPs as in Jin-Cheng et al. [12] to screen for those genes that were significantly associated with OS in the training data set, with *p* < 0.05 as the threshold.

### Screening for robust immune-related prognostic features

LASSO is a popular method for regression modeling with a large number of potential prognostic features, because it can perform automatic feature selection in a manner that results in signatures with generally good prognostic performance [13]. The LASSO method has been extended to the Cox model for survival analysis and has been successfully applied for the purpose of building sparse signatures for survival prognosis in many application areas including oncology [14–16], First, we used training set samples to conduct univariate Cox proportional risk regression analysis for each IRGPs, with log rank *p* < 0.05 as the threshold, 669 IRGPs with significantly correlated prognoses were identified. Furthermore, R software package glmnet [17] was used to screen robust prognostic immune-related gene pairs, and 3-fold cross validation was used to evaluate the optimal characteristics. The degree of LASSO regression complexity adjustment is controlled by the parameter λ, where the larger λ is, the greater the penalty for a linear model with more variables, so that a model with fewer variables is eventually obtained. In this study, the optimal model is obtained when λ = 0.1218186, and we choose the features incorporated in the model at this time as the optimal combination of features, i.e., 14-IRGPs. Multivariate Cox regression analysis was conducted using the stepwise regression method to determine the coefficient of each IRGPs in the 14-IRGPs, and the following risk score model was constructed:

$$ RiskScore=\sum \limits_{k=1}^n{Exp}_k\ast {e^{HR}}_k $$

Where N is the number of prognostic IRGPs, *Exp*_*k*_ is the IRGP value of prognostic IRGPs, and *e*^*HR*^_*k*_ is the estimated regression coefficient of IRGPs in the multivariate Cox regression analysis.

### Validation and assessment of the IRGPs signature

To validate the IRGPs signature, patients in test datasets were divided into low risk and high risk group according to the median value of the risk score, which calculated according to the prognostic signature. The log-rank test and Cox regression analysis were conducted to evaluate overall survival difference between the low risk and high risk groups. Receiver operating characteristic curve (ROC) curve was used to assess the categorization of IRGPs signature. The IRGPs signature was also compared with the published signature by KM survival curve, ROC curve, and C-index.

### RiskScore and clinical characteristics

In order to observe the relationship between riskScore and clinical phenotype, the samples were divided into two groups based on the riskScore median of the samples, and the prognosis differences between high riskScore and low riskScore were compared respectively. Similarly, the relationship Grade, Age and Stage in High and Low TMEScore was analyzed.

### Functional enrichment analysis

We used R package clusterProfiler, v3.8 [18] for GO and KEGG enrichment analysis with a *p* value of less than 0.05 as the threshold. GSEA [19] was performed by R package GSVA using the MSigDB [20]. Gene sets with a false discovery rate (FDR) value less than 0.05 after performing 1000 permutations were considered to be significantly enriched.

### Statistical analysis

The Kaplan-Meier (KM) curve was plotted when the median risk score in each data set was used as a cutoff to compare the risk of survival between the high risk group and the low risk group. Multivariate Cox regression analysis was performed to test whether gene markers are independent prognostic factors. Significance was defined as *P* < 0.05. AUC analysis was performed using the R package pROC. All analyses use default parameters except for special instructions, which are performed in R software version 3.4.3.

## Results

### Identification of IRGPs in patients with HNSCC

**Fig. 2:**
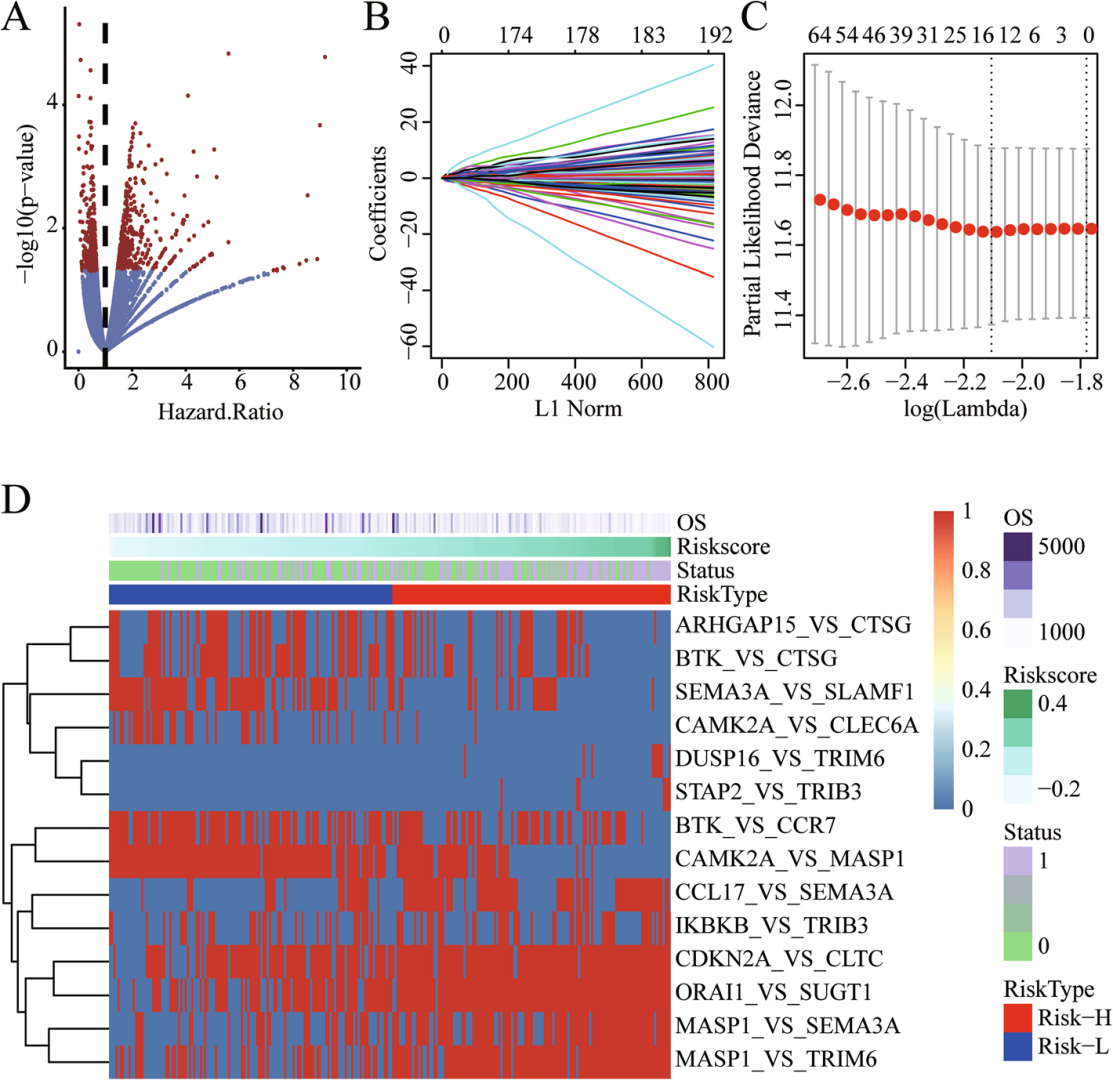
**a**: The relationship between the *P* value of 699 IRGPs and HR. Red indicated log rank *p* < 0.05 IRGPs. **b**: The trajectory of each independent variable, the horizontal axis represents the log value of the independent variable lambda, and the vertical axis represents the coefficient of the independent variable. **c**: The confidence interval under each lambda. **d**: Relationship between 14 IRGPs and risk scores

For the TCGA training set samples, we used a univariate Cox proportional hazard regression model to establish the relationship between patient overall survival and immune-related gene expression, and obtained 164 prognostic genes. According to the calculation rule of the IRGPs value, a total of 7374 IRGPs are obtained. The univariate Cox proportional hazards regression model was used to establish the relationship between IRGPs and overall patient survival. Finally, we obtained 699 IRGPs with significant prognostic differences (Fig. 2a). In order to screen robust immune-related prognostic gene pairs, we used lasso regression to perform dimensionality reduction analysis on these 699 IRGPs. The results show that as the lambda increases, the number of independent coefficients tends to 0 (Fig. 2b), 3-fold cross-validation was used to build the model, and the model is optimal when lambda = 0.1218186 (Fig. 2c). We select the model when lambda = 0.1218186 as the final model, which contains a total of 14 IRGPs, 19 genes (Table 3). The risk scores of these 14 IRGPs in each sample are shown in Fig. 2d. Furthermore, we calculated the Risk Score of each sample based on the Risk model, and the formula is as follows:

$$ \mathrm{RiskScore}14=-0.0415\ast \mathrm{ARHGAP}15\_\mathrm{VS}\_\mathrm{CTSG}-0.0015\ast \mathrm{BTK}\_\mathrm{VS}\_\mathrm{CCR}7-0.0371\ast \mathrm{BTK}\_\mathrm{VS}\_\mathrm{CTSG}-0.0728\ast \mathrm{CAMK}2\mathrm{A}\_\mathrm{VS}\_\mathrm{CLEC}6\mathrm{A}-0.1534\ast \mathrm{CAMK}2\mathrm{A}\_\mathrm{VS}\_\mathrm{MASP}1+0.0241\ast \mathrm{CCL}17\_\mathrm{VS}\_\mathrm{SEMA}3\mathrm{A}+0.1431\ast \mathrm{CDKN}2\mathrm{A}\_\mathrm{VS}\_\mathrm{CLTC}+0.1202\ast \mathrm{DUSP}16\_\mathrm{VS}\_\mathrm{TRIM}6+0.0069\ast \mathrm{IKBKB}\_\mathrm{VS}\_\mathrm{TRIB}3+0.00325\ast \mathrm{MASP}1\_\mathrm{VS}\_\mathrm{SEMA}3\mathrm{A}+0.0727\ast \mathrm{MASP}1\_\mathrm{VS}\_\mathrm{TRIM}6+0.0751\ast \mathrm{ORAI}1\_\mathrm{VS}\_\mathrm{SUGT}1-0.0662\ast \mathrm{SEMA}3\mathrm{A}\_\mathrm{VS}\_\mathrm{SLAMF}1+0.1548\ast \mathrm{STAP}2\_\mathrm{VS}\_\mathrm{TRIB}3 $$

**Table 3:** 14 IRGPs associated with prognosis

IRGPs	Coef	***P*** value	HR	Low.95.CI.	High.95.CI.
ARHGAP15_VS_CTSG	−0.0415	0.001833936	0.507	0.331	0.778
BTK_VS_CCR7	−0.0015	0.000471643	0.512	0.352	0.745
BTK_VS_CTSG	−0.0371	0.00101336	0.512	0.343	0.763
CAMK2A_VS_CLEC6A	−0.0728	0.001000155	0.335	0.175	0.642
CAMK2A_VS_MASP1	−0.1535	2.82E-05	0.452	0.312	0.656
CCL17_VS_SEMA3A	0.0242	0.00023524	2.019	1.388	2.935
CDKN2A_VS_CLTC	0.1432	0.000924265	2.866	1.537	5.344
DUSP16_VS_TRIM6	0.1202	7.15E-05	4.076	2.037	8.154
IKBKB_VS_TRIB3	0.007	0.001015171	1.879	1.290	2.738
MASP1_VS_SEMA3A	0.0033	0.000200249	2.122	1.427	3.154
MASP1_VS_TRIM6	0.0728	0.000288898	2.307	1.468	3.626
ORAI1_VS_SUGT1	0.0751	0.000457145	2.617	1.528	4.481
SEMA3A_VS_SLAMF1	−0.0663	0.000190953	0.459	0.305	0.691
STAP2_VS_TRIB3	0.1549	1.47E-05	5.593	2.567	12.186

### 14-IRGPs signature could be used as a prognostic marker

**Fig. 3:**
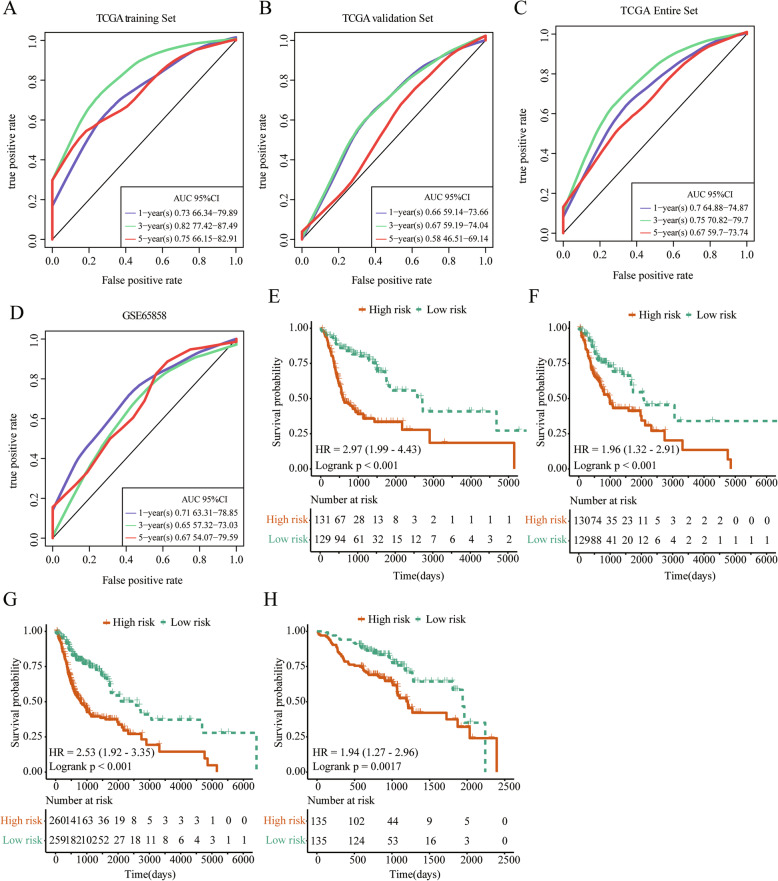
**a**: The risk model ROC of 14 IRGPs after lasso regression in TCGA training dataset. **b**: The risk model ROC of 14 IRGPs after lasso regression in TCGA validation dataset. **c**: The risk model ROC of 14 IRGPs after lasso regression in TCGA dataset. **d**: The risk model ROC of 14 IRGPs after lasso regression in GSE65858 dataset. **e**: KM survival curve of 14 IRGPs in TCGA training dataset. **f**: KM survival curve of 14 IRGPs in TCGA validation dataset. **g**: KM survival curve of 14 IRGPs in TCGA dataset. **h**: KM survival curve of 14 IRGPs in GSE65858 dataset

Multivariate regression analysis was used to establish a risk model for 14 IRGPs in the training set, validation set, TCGA dataset and independent test set data (GSE65858 dataset) for 1, 3, and 5 years. The results suggest that the average AUC of the training set is 0.758, the average AUC of the validation set is 0.659, the average AUC of the TCGA dataset is 0.709, and the average AUC of the independent test set data is 0.685 (Fig. 3a-d). With the median risk score as the threshold, the training set samples were divided into risk-H and risk-L, and the KM survival curves of 14-IRGPs in training set, validation set, all data sets of TCGA and one independent GEO testsets (GSE65858 dataset) were drawn. The results showed that the prognosis of the risk-L group of all data sets was significantly better than that of the risk-H group (Fig. 3e-h). In summary, IRGPs have great potential as prognostic markers.

### Predictive power of risk models in different clinical samples

**Fig. 4:**
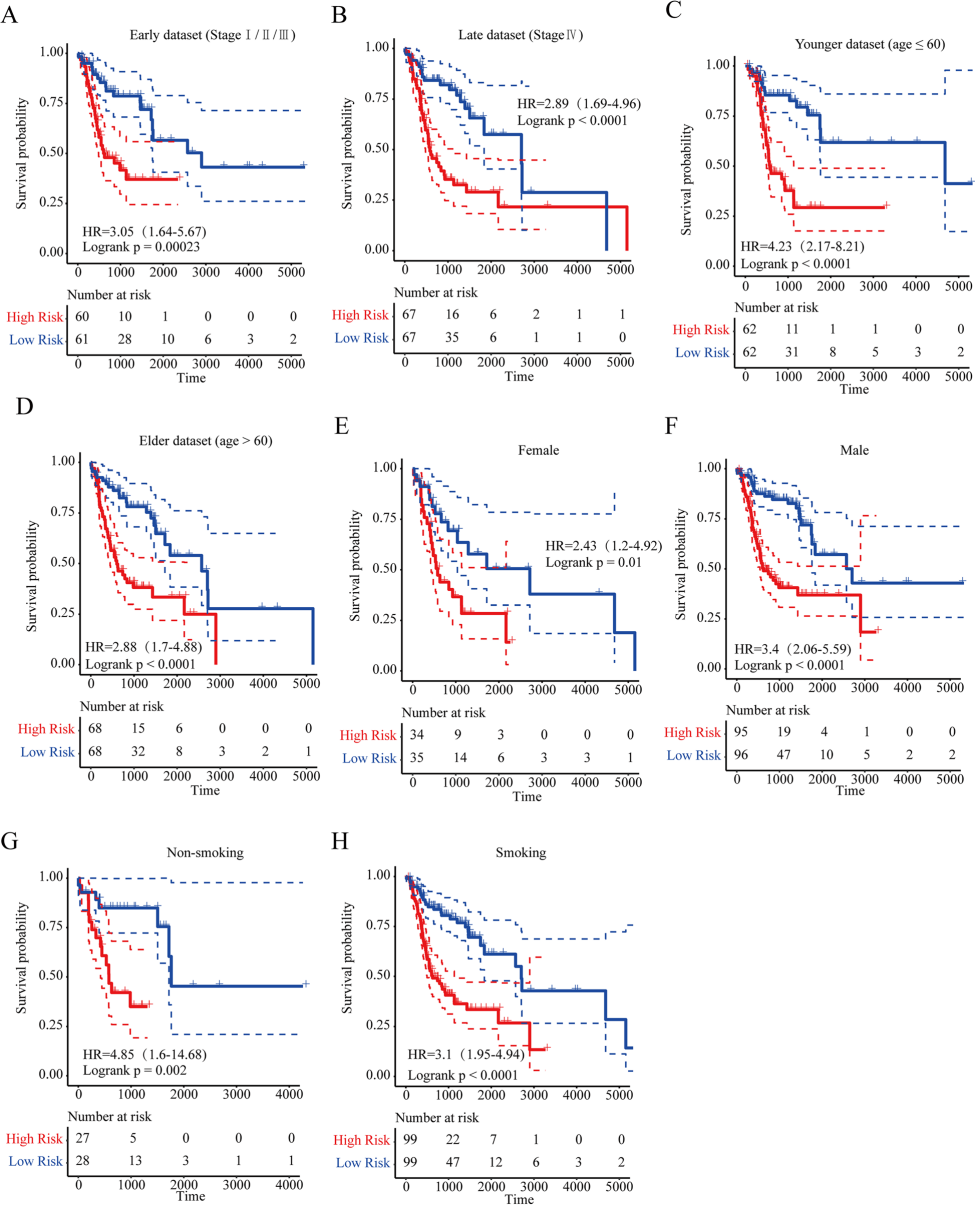
**a**: KM curve of 14-IRGPs signature in early samples. **b**: KM curve of 14-IRGPs signature in advanced cancer sample. **c**: KM curve of 14-IRGPs signature in young. **d**: KM curve of 14-IRGPs signature in age. **e**: KM curve of 14-IRGPs signature in female. **f**: KM curve of 14-IRGPs signature in male. **g**: KM curve of 14-IRGPs signature in No smoking history sample. **h**: KM curve of 14-IRGPs signature in smoking history sample

In order to observe the robustness of risk models in different clinical characteristics, we observed the predictive power of risk models in different TNMstages, Age, gender and smoking history. We found that the 14-IRGPs signature model can be significantly distinguished into high-risk group and low-risk group not only in early patients and late-stage patients (Fig. 4a, b) (log rank *p* = 0.00023, log rank *p* < 0.0001), but also in young data sets and elderly data sets (Fig. 4c, d) (logrank *p* < 0.0001, log rank *p* < 0.0001), and in female data sets and male data sets (Fig. 4e, f) (log rank *p* = 0.01, log rank *p* < 0.0001). Finally, our analysis of the samples with and without smoking history shows that 14-IRGPs signature can also significantly distinguish the high-risk group from the low-risk group (Fig. 4g, h) (log rank *p* = 0.002, log rank *p* < 0.0001), those results indicated that our model has a very stable predictive power in patients of different ages, stages and genders.

### Univariate and multivariate analysis of 14-IRGPs signature

In order to identify the independence of 14-IRGPs signature model in clinical application, we used univariate and multivariate COX regression analysis to analyze relevant HR, 95%CI of HR, *p* value in TCGA training set, TCGA verification data set and all data of TCGA. We systematically analyzed clinical information recorded by TCGA patients, including age, T, N, AJCC Stage, Grade, Smoking, and our 14-IRGPs signature grouping information (Table 4).

**Table 4:** Univariate and multivariate COX regression analysis identified clinical factors associated with prognosis

Variables	Univariate analysis			Multivariable analysis		
	HR	95%CI of HR	***P*** value	HR	95%CI of HR	***P*** value
**TCGA training datasets**						
14-IRGPs signature						
Risk score (High/Low)	2.97	1.99–4.43	7.79E-08	2.53	1.54–4.13	0.0002
Age	1.02	1.01–1.04	0.009	1.02	0.99–1.04	0.0550
Gender (Male vs Female)	0.70	0.47–1.04	0.077	0.94	0.56–1.5	0.8236
T3/T4 vs T1/T2	0.97	0.66–1.43	0.895	0.43	0.24–0.74	0.0025
N1/N2/N3 VS N0	1.21	0.78–1.85	0.39	1.00	0.59–1.68	0.9985
Stage IV vs Stage I/ II/III	1.97	1.31–2.95	0.001	2.65	1.46–4.78	0.0012
G3/G4 vs G1/G2	1.16	0.76–1.77	0.491	0.94	0.57–1.52	0.7969
Smoking vs Non-smoking	0.95	0.59–1.52	8.35E-01	0.85	0.48–1.49	0.5707
**TCGA validation datasets**						
14-IRGPs signature						
Risk score (High/Low)	1.96	1.32–2.91	7.93E-04	1.72	1.12–2.62	0.0123
Age	1.02	0.99–1.04	0.064	1.02	0.99–1.03	0.1549
Gender (Male vs Female)	0.77	0.51–1.17	0.223	0.79	0.47–1.31	0.3577
T3/T4 vs T1/T2	1.75	1.13–2.71	0.011	2.05	1.17–3.59	0.0120
N1/N2/N3 VS N0	1.29	0.87–1.91	0.209	1.39	0.86–2.23	0.1674
Stage IV vs Stage I/ II/III	1.36	0.90–2.05	0.142	0.87	0.49–1.53	0.6379
G3/G4 vs G1/G2	1.22	0.77–1.93	0.393	1.20	0.74–1.93	0.4439
Smoking vs Non-smoking	1.33	0.82–2.15	0.247	1.34	0.77–2.31	0.2987
**TCGA entire datasets**						
14-IRGPs signature						
Risk score (High/Low)	2.54	1.92–3.35	6.51E-11	2.01	1.41–2.84	9.36E-05
Age	1.02	1.01–1.03	0.0013	1.02	1.01–1.04	0.0087
Gender (Male vs Female)	0.74	0.55–0.97	0.034	0.97	0.66–1.41	0.8830
T3/T4 vs T1/T2	1.28	0.96–1.70	0.093	0.72	0.47–1.09	0.1107
N1/N2/N3 VS N0	1.20	0.87–1.66	0.271	1.07	0.73–1.54	0.7276
Stage IV vs Stage I/ II/III	1.65	1.24–2.20	6.20E-04	1.85	1.21–2.82	0.0046
G3/G4 vs G1/G2	1.15	0.84–1.57	0.365	0.83	0.58–1.18	0.3067
Smoking vs Non-smoking	1.14	0.81–1.59	0.45	1.07	0.7–1.63	0.7539
**GSE65858**						
14-IRGPs signature						
Risk score (High/Low)	1.94	1.27–2.97	0.0021	1.90	1.23–2.92	0.0035
Age	1.03	1.01–1.05	0.0130	1.04	1.01–1.06	0.0036
Gender (Male vs Female)	1.05	0.62–1.77	0.8680	1.06	0.62–1.82	0.8378
T3/T4 vs T1/T2	2.92	1.81–4.72	1.23E-05	2.16	1.26–3.69	0.0049
N1/N2/N3 VS N0	2.14	1.38–3.32	0.0007	1.39	0.73–2.65	0.3216
Stage IV vs Stage I/ II/III	2.92	1.72–4.95	7.22E-05	1.55	0.68–3.55	0.2987
Smoking vs Non-smoking	0.94	0.55–1.59	0.8210	1.14	0.64–2.03	0.6493

In the training set of TCGA, univariate COX regression analysis found that Risk score, AJCC Stage and Smoking were significantly correlated with survival, but the corresponding multi-factor COX regression analysis found that Risk score (HR = 2.53, 95%CI = 1.54–4.13, *p* = 0.0002), T Stage and AJCC Stage were significantly correlated with survival.

In the verification set of TCGA, univariate COX regression analysis found that Risk score and T staging were significantly correlated with survival, but the corresponding multivariate COX regression analysis found that Risk score (HR = 1.72, 95%CI = 1.12–2.62, *p* = 0.0123) and T staging were significantly correlated with survival.

In all data sets of TCGA, univariate COX regression analysis found that Risk score, age, gender and AJCC stage were significantly correlated with survival, but the corresponding multivariate COX regression analysis found that Risk score (HR = 2.01, 95%CI = 1.41–2.84, *p* < 0.0001), age and AJCC stage staging were significantly correlated with survival.

Finally, in the GEO external data set, univariate COX regression analysis found that Risk score, age, T stage, N stage and AJCC stage were significantly correlated with survival, but the corresponding multivariate COX regression analysis found that Risk score (HR = 1.90, 95%CI = 1.23–2.92, *p* = 0.0035), age and T stage were significantly correlated with survival.

The above conditions indicate that our model 14-IRGPs signature has a good predictive performance in terms of clinical application value in TCGA data set, and our model may be a prognostic indicator independent of other clinical factors and has an independent predictive performance in terms of clinical application value.

### Functional analysis and immune analysis of IRGPs

**Fig. 5:**
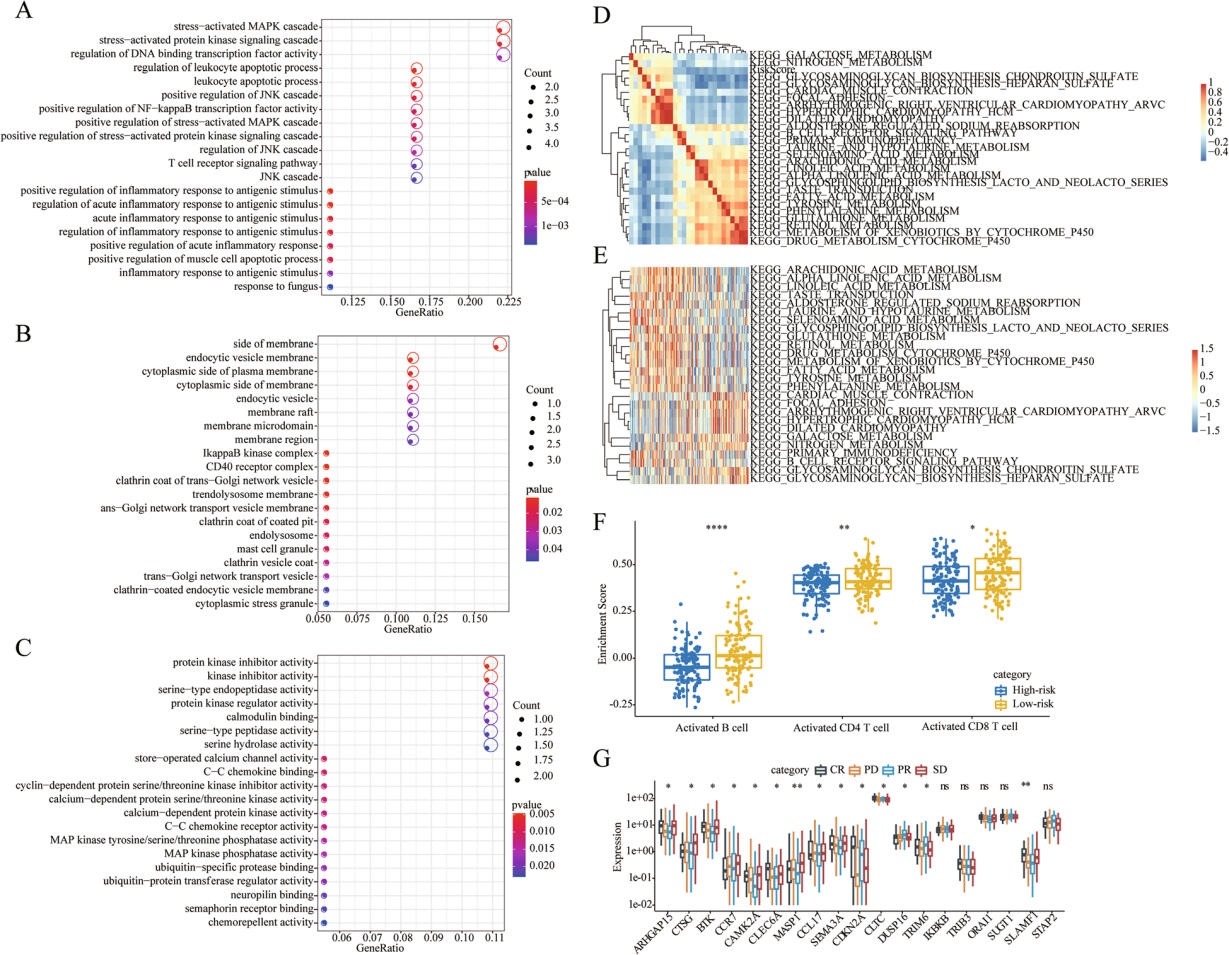
**a**: Top 20 GO BP enrichment results of 19 immune-related genes. **b**: Top 20 GO CC enrichment results for 19 immune-related genes. **c**: Top 20 GO MF enrichment results for 19 immune-related genes. **d**: Clustering of correlation coefficients between KEGG pathways and RiskScores with a risk score correlation > 0.25. **e**: The KEGG pathway with a correlation with risk scores greater than 0.25 has a relationship with the ssGSEA score in each sample as the risk score increases. The horizontal axis represents the sample, and the risk score from left to right increases in turn. **f**: The difference of B cell and T cell activation scores between high and low risk groups. **g**: Expression differences of 19 genes in 14-IRGPs in patients with different response states after PD-L1 treatment; CR: complete response, PD: progressive disease, SD: stable disease, PR: partial response

In order to further analyze the functions of 14-IRGPs, we first used clusterProfiler to conduct GO and KEGG enrichment analysis on 19 genes, and finally retained the results of *p* < 0.05. The results showed that these pathways were enriched to 356 GO BP, which were mainly T cell receptor signaling pathway, stress-activated MAPK cascade and other biological processes, and we show the most significant top 20 (Fig. 5a), Furthermore, we found that these 19 genes were significantly enriched in 39 GO CCs and 73 GO MFs, the most prominent of which were the top 20 (Fig. 5b, c).

ssGSEA was used to analyze the enrichment scores of each sample in each pathway in the TCGA data set, calculate the correlation between these pathways and risk scores, and 26 pathways with correlation > 0.25 were selected (Fig. 5d), we found that most of the samples with risk score present negative correlation, a small number of positively related with risk score. Cluster analysis was conducted according to the 26 KEGG pathway enrichment scores (Fig. 5e), it can be seen that among the 26 pathways, B CELL SIGNALING PATHWAY, PRIMARY IMMUNODEFICIENCY and other pathways increase with the increase of RiskScore, and FOCAL ADHESION, GALACTOSE_METABOLISM and other metabolism-related pathways decrease with the increase of RiskScore. Results suggested that the imbalance of these pathways is closely related to the development of tumors.

In addition, we obtained the signature genes of three immune cells, Activated B cell, Activated CD4 T cell, and Activated CD8 T cell, from a previous study [21], and calculated enrichment scores in each sample using the method of ssGSEA to assess the sample’s corresponding Immune cell scores. The differences in these three immune cell scores in the high and low risk groups of patients were analyzed and observed that B cell and T cell activation scores were all significantly lower in high risk patients with poor prognosis (Fig. 5f). Current immunotherapy-related datasets are rare. We found a cohort of PD-L1-treated patients with metastatic uroepithelial carcinoma shared by Sanjeev Mariathasan et al [22] and analyzed the differential expression of 19 genes in 14 IRGPs in patients with different response states after PD-L1 treatment. We observed a significant differential expression of 14 (73.6%) genes (Fig. 5g), suggesting that these genes are associated with immunotherapy.

### Establishment and evaluation of nomogram model

**Fig. 6:**
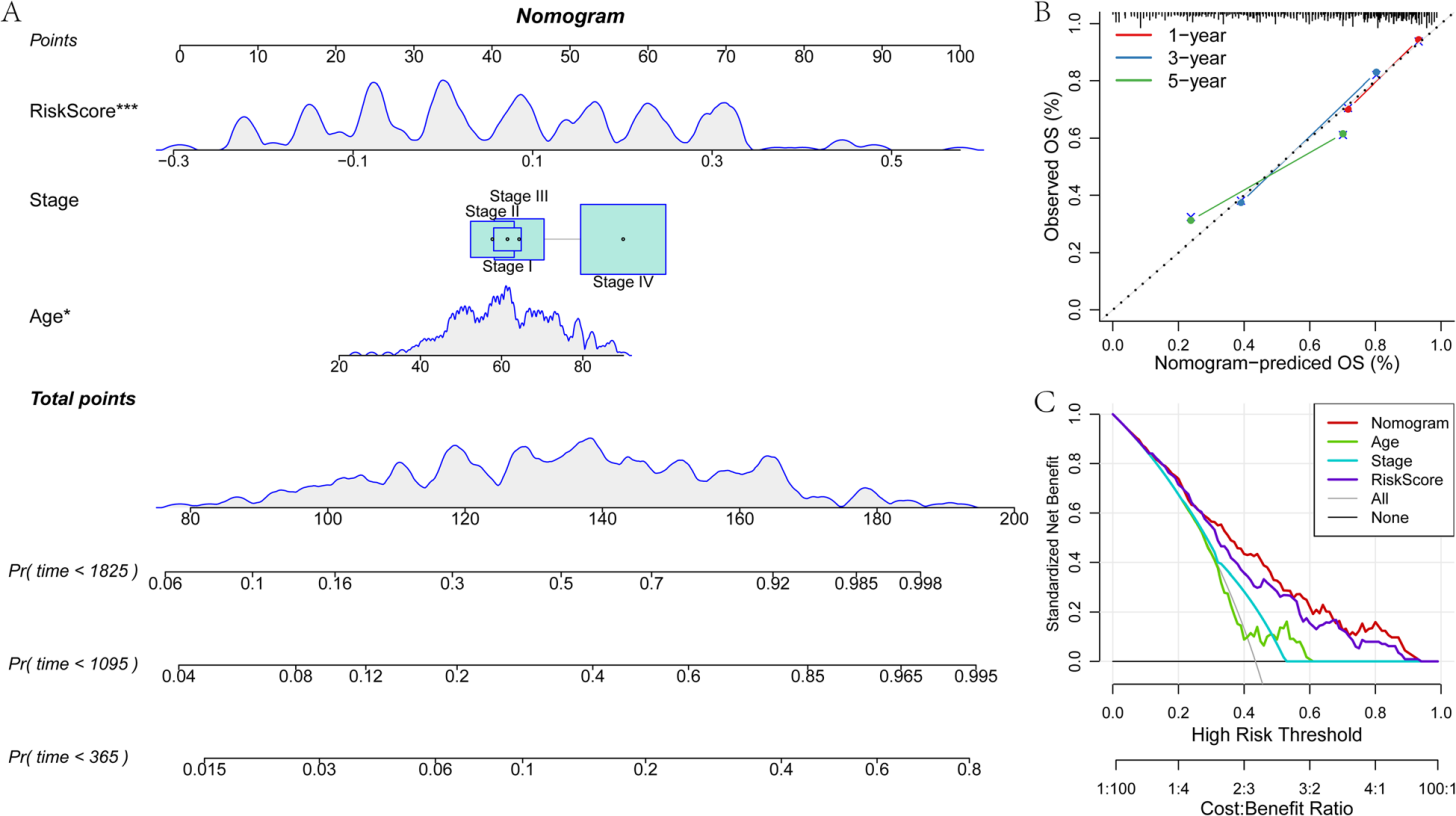
Establishment and evaluation of nomogram model. **a**: The nomogram model combined with Stage, Age and 14-IRGPs. **b**: Calibration curves of the nomogram for 1, 3 and 5 years. **c**: Decision curves for Stages, Ages, 14-IRGPs and nomograms

In addition to 14-IRGPs, clinical features Stage and Age are also independent prognostic factors, indicating that they have complementary values. In order to further improve the accuracy of prediction, a new nomogram was established by integrating Stage, Age and 14-IRGPs using Cox model. According to this model, 14-IRGPs contribute the most to OS, followed by Age and Stage (Fig. 6a). By calculating the total score, oncologists could easily obtain the OS probability predicted by the nomogram of an individual patient. Furthermore, we used the calibration curve to evaluate the prediction accuracy of the model (Fig. 6b), results show that the predicted calibration curves of the three calibration points in 1, 3, and 5 years were close to the standard curve, which indicated that the model has good prediction performance. In addition, we also used DCA (Decision curve) to evaluate the reliability of the model (Fig. 6c). It was observed that RiskScore (14-IRGPs) and nomogram benefit significantly higher than the extreme curve, and nomogram is higher than RiskScore, and Age and Stage are close to the extreme curve. This suggests that RiskScore (14-IRGPs) and nomogram have good reliability.

### 14-IRGPs was compared with other signatures and clinical features

In order to observe the performance of 14-IRGPs, the prognostic signature of three head and neck cancers reported in the past (3-gene signature of Cui L et al [23], 6-gene signature of Weidong Zhang et al [24] and 3-gene signature of Hongbo Zhou et al [25]) and four clinical features of T, N, age and Stage were selected. In order to make the model comparable, we calculated the Risk score of each head and neck cancer sample in the TCGA training set with the same method according to the corresponding genes in the 3 models, evaluated the ROC of each model and C-index (Table 5). We observe that the 6-gene signature model has the highest AUC above among the three models, while the average AUC for 1, 3 and 5 years is 0.63. The 1, 3, and 5 years AUC of 14-IRGPs signature were all above 0.73. In addition, in the C-index of all models, 14-IRGPs was significantly higher than other clinical features and models, indicating that our model has good application value.

**Table 5:** Comparision for 4 models and clinical features

Characteristics	C-index (95%CI)	1-year AUC (95%CI)	3-year AUC (95%CI)	5-year AUC (95%CI)
T	0.50 (0.426–0.583, 0.571)	0.53 (0.47–0.6)	0.52 (0.46–0.58)	0.6 (0.52–0.67)
N	0.53 (0.452–0.610,0.835)	0.52 (0.47–0.59)	0.53 (0.47–0.59)	0.45 (0.46–0.54)
Age	0.56 (0.490–0.626,0.0096)	0.62 (0.53–0.71)	0.55 (0.48–0.62)	0.51 (0.42–0.6)
AJCC stage	0.52 (0.434–0.600,0.412)	0.56 (0.5–0.63)	0.58 (0.52–0.64)	0.5 (0.41–0.59)
14-IGPS signature	0.78 (0.693–0.859,3.65E-08)	0.73 (0.66–0.8)	0.82 (0.77–0.87)	0.75 (0.66–0.82)
3-gene signature	0.67 (0.579–0.767, 0.002)	0.56 (0.48–0.63)	0.64 (0.57–0.71)	0.63 (0.52–0.74)
6-gene signature	0.65 (0.560–0.750, 7.07E-5)	0.56 (0.48–0.63)	0.63 (0.56–0.7)	0.71 (0.62–0.81)
3-gene signature	0.62 (0.519–0.715,0.043	0.59 (0.50–0.67)	0.58 (0.51–0.66)	0.51 (0.40–0.61)

## Discussion

Due to the heterogeneity of HNSCC, patients are still at great risk of recurrence and death even after complete surgical resection. The management of adjuvant chemotherapy for early HNSCC remains controversial. Therefore, it is important to develop a personalized management approach for HNSCC. Reliable prognostic biomarkers can identify patients with poor prognosis, and predictive biomarkers can inform patients who may benefit from additional systemic therapy, regardless of treatment, and therefore have more direct clinical relevance. In this study, we developed immune-related genes for signature prediction of HNSCC prognosis. Their potential for molecular stratification of HNSCC suggests different immune characteristics at different stages of the tumor.

In the past decade, important studies based on prognostic signals of immune gene expression have shown that immune genes have a strong prognostic ability. Several gene expression scores have been proposed for predicting the risk of recurrence, and both Tadalafil and anti-tumor vaccine-mediated immune rejection reversals also lead to up-regulation of PDL1 in recurrent HNSCC, suggesting that immune checkpoint therapy may be effective in patients with HNSCC [8]. Immune-related gene signature reflecting immune infiltration can predict the prognosis of colorectal cancer [26]. AP001056.1 is a key immune-related ceRNA in SCCHN, and ICOSLG encodes immune checkpoint protein as its regulatory target, which can be used as a prognostic molecule of HNSCC [27]. The 14-IRGPs we developed could be risk stratified in four data sets, with AUC higher than 0.659. The KM curve of the risk score in the four data sets indicates that high risk predicts poor prognosis, and those results indicated that the immune-related gene can be used as a factor for stratifying the prognosis risk of HNSCC.

In order to observe whether 14-IRGPs signature is dependent on TP53 and EGFR mutation characteristics, we first compared the relationship among 14-IRGPs signature, TP53 and EGFR mutation using single-factor and multi-factor analysis (Figure S1A-B). The results showed that 14-IRGPs signature had significant difference in prognosis, suggesting that 14-IRGPs signature is an independent factor. Furthermore, we compared the ROC analysis of 14-IRGPs signature in mutant and non-mutant samples and, considering the small number of EGFR mutations, only TP53 mutations were analyzed here (Figure S1C-D). We observed that the 14-IRGPs signature had higher AUC in both TP53 mutant and non-mutant samples. We also observed the lowest AUC at 1 year in TP53 mutant samples and the lowest AUC at 5 years in non-mutated samples, suggesting that the 14-IRGPs signature has better predictive performance for long-term survival in TP53 mutant samples and for short-term survival in non-mutated samples. We downloaded exon datasets of TCGA samples and extracted mutation data from HNSCC samples, in which a total of 508 patients were tested. Nineteen genes in the 14-IRGPs signature were analyzed for their mutation frequencies in these patients (Figure S1E), which had the highest frequency of CDKN2A mutations, especially in high-risk patients, mainly Nonsense_Mutation.

Go and KEGG analysis were conducted to identify the functions of the 19 genes involved in HNSCC. T cell receptor signaling pathway, stress-activated MAPK cascade, B cell signaling pathway and primary immunodeficiency were enriched in TCGA samples. These immune-related pathways are involved in various biological processes, such as differentiation, growth, and apoptosis, and promote cell interaction and migration [28, 29]. Taken together, those pathways may facilitate the metastasis of HNSCC.

Comprehensive analysis shows that risk score is a prognostic biomarker for HNSCC and can be used to molecularly stratify prognosis. Clinical features Age, Stage and Grade are key prognostic factors in head and neck squamous cell carcinoma. Factor [30], As expected, there is a significant association between the risk score and Age, Stage, and Grade, and found that the combination of risk score and Age has a superior prognostic effect.

Although we identify potential candidate IRGPs involved in tumorigenesis in large samples by bioinformatics techniques, some limitations of this study should be noted. First, the sample lacks some clinical follow-up information, so we did not consider factors such as the presence of other health status of the patient to distinguish prognostic biomarkers. Second, the results obtained only through bioinformatics analysis are inadequate and experimental validation is needed to confirm these results. Therefore, further genetic and experimental studies of larger sample sizes and experimental validation are needed.

## Conclusions

In conclusion, we studied the immunological characteristics of HNSCC and systematically studied the expression profile of immune genes. We found immune-related gene pair features in HNSCC, and have better AUC in both training and validation sets. Compared with clinical features, immune gene pair classifiers could improve survival risk prediction. Therefore, we recommend using this classifier as a molecular diagnostic test to assess the prognostic risk of patients with HNSCC.

## Supplementary information

**Additional file 1.**

## Data Availability

The datasets used and analysed during the current study are available from the corresponding author on reasonable request.

## Abbreviations

HNSCC: Head and neck squamous cell carcinoma
TCGA: The Cancer Genome Atlas
IRGPs: Immune-related gene pairs
HPV: Human papillomavirus
FDA: Food and drug administration
FDR: False discovery rate
ssGSEA: Single sample gene set enrichment analysis
KM: Kaplan-Meier

## References

[1] Siegel R, Naishadham D, Jemal A (2012). Cancer statistics, 2012. CA Cancer J Clin.

[2] Rautava J, Syrjanen S (2012). Biology of human papillomavirus infections in head and neck carcinogenesis. Head Neck Pathol.

[3] Posner M, Vermorken JB (2008). Induction therapy in the modern era of combined-modality therapy for locally advanced head and neck cancer. Semin Oncol.

[4] Kobold S, Pantelyushin S, Rataj F, Vom Berg J (2018). Rationale for combining Bispecific T cell activating antibodies with checkpoint blockade for cancer therapy. Front Oncol.

[5] Popovic A, Jaffee EM, Zaidi N (2018). Emerging strategies for combination checkpoint modulators in cancer immunotherapy. J Clin Invest.

[6] Li S, Yang F, Ren X (2015). Immunotherapy for hepatocellular carcinoma. Drug Discov Ther.

[7] Forster MD, Devlin MJ (2018). Immune checkpoint inhibition in head and neck cancer. Front Oncol.

[8] Weed DT, Zilio S, Reis IM, Sargi Z, Abouyared M, Gomez-Fernandez CR, Civantos FJ, Rodriguez CP, Serafini P (2019). The reversal of immune exclusion mediated by Tadalafil and an anti-tumor vaccine also induces PDL1 upregulation in recurrent head and neck squamous cell carcinoma: interim analysis of a phase I clinical trial. Front Immunol.

[9] Cohen EEW, Bell RB, Bifulco CB, Burtness B, Gillison ML, Harrington KJ, Le QT, Lee NY, Leidner R, Lewis RL (2019). The Society for Immunotherapy of Cancer consensus statement on immunotherapy for the treatment of squamous cell carcinoma of the head and neck (HNSCC). J Immunother Cancer.

[10] Han J, Chen M, Wang Y, Gong B, Zhuang T, Liang L, Qiao H (2018). Identification of biomarkers based on differentially expressed genes in papillary thyroid carcinoma. Sci Rep.

[11] Li B, Cui Y, Diehn M, Li R (2017). Development and validation of an individualized immune prognostic signature in early-stage nonsquamous non-small cell lung cancer. JAMA Oncol.

[12] Yuan L, Guo LH, Yuan CA, Zhang YH, Han K, Nandi A, Honig B, Huang DS (2018). Integration of multi-omics data for gene regulatory network inference and application to breast cancer. IEEE/ACM Trans Comput Biol Bioinformatics.

[13] Kostareli E, Hielscher T, Zucknick M, Baboci L, Wichmann G, Holzinger D, Mucke O, Pawlita M, Del Mistro A, Boscolo-Rizzo P (2016). Gene promoter methylation signature predicts survival of head and neck squamous cell carcinoma patients. Epigenetics.

[14] Zhang JX, Song W, Chen ZH, Wei JH, Liao YJ, Lei J, Hu M, Chen GZ, Liao B, Lu J (2013). Prognostic and predictive value of a microRNA signature in stage II colon cancer: a microRNA expression analysis. Lancet Oncol.

[15] Papaemmanuil E, Gerstung M, Malcovati L, Tauro S, Gundem G, Van Loo P, Yoon CJ, Ellis P, Wedge DC, Pellagatti A (2013). Clinical and biological implications of driver mutations in myelodysplastic syndromes. Blood.

[16] Yuan Y, Van Allen EM, Omberg L, Wagle N, Amin-Mansour A, Sokolov A, Byers LA, Xu Y, Hess KR, Diao L (2014). Assessing the clinical utility of cancer genomic and proteomic data across tumor types. Nat Biotechnol.

[17] Friedman J, Hastie T, Tibshirani R (2010). Regularization paths for generalized linear models via coordinate descent. J Stat Softw.

[18] Sherman BT, Huang d W, Tan Q, Guo Y, Bour S, Liu D, Stephens R, Baseler MW, Lane HC, Lempicki RA (2007). DAVID Knowledgebase: a gene-centered database integrating heterogeneous gene annotation resources to facilitate high-throughput gene functional analysis. BMC Bioinformatics.

[19] Subramanian A, Kuehn H, Gould J, Tamayo P, Mesirov JP (2007). GSEA-P: a desktop application for gene set enrichment analysis. Bioinformatics.

[20] Liberzon A, Subramanian A, Pinchback R, Thorvaldsdottir H, Tamayo P, Mesirov JP (2011). Molecular signatures database (MSigDB) 3.0. Bioinformatics.

[21] Charoentong P, Finotello F, Angelova M, Mayer C, Efremova M, Rieder D, Hackl H, Trajanoski Z (2017). Pan-cancer immunogenomic analyses reveal genotype-immunophenotype relationships and predictors of response to checkpoint blockade. Cell Rep.

[22] Mariathasan S, Turley SJ, Nickles D, Castiglioni A, Yuen K, Wang Y, Kadel EE, Koeppen H, Astarita JL, Cubas R (2018). TGFβ attenuates tumour response to PD-L1 blockade by contributing to exclusion of T cells. Nature.

[23] Zhao X, Sun S, Zeng X, Cui L (2018). Expression profiles analysis identifies a novel three-mRNA signature to predict overall survival in oral squamous cell carcinoma. Am J Cancer Res.

[24] Tian S, Meng G, Zhang W (2019). A six-mRNA prognostic model to predict survival in head and neck squamous cell carcinoma. Cancer Manag Res.

[25] Cao R, Wu Q, Li Q, Yao M, Zhou H (2019). A 3-mRNA-based prognostic signature of survival in oral squamous cell carcinoma. PeerJ.

[26] Wu J, Zhao Y, Zhang J, Wu Q, Wang W (2019). Development and validation of an immune-related gene pairs signature in colorectal cancer. Oncoimmunology.

[27] Gu X, Wang L, Boldrup L, Coates PJ, Fahraeus R, Sgaramella N, Wilms T, Nylander K (2019). AP001056.1, a prognosis-related enhancer RNA in squamous cell carcinoma of the head and neck. Cancers.

[28] Chen L, Diao L, Yang Y, Yi X, Rodriguez BL, Li Y, Villalobos PA, Cascone T, Liu X, Tan L (2018). CD38-mediated immunosuppression as a mechanism of tumor cell escape from PD-1/PD-L1 blockade. Cancer Discov.

[29] Wang Q, Li P, Wu W (2019). A systematic analysis of immune genes and overall survival in cancer patients. BMC Cancer.

[30] Song MJ, Lim SY, Park JS, Yoon HI, Lee JH, Kim SY, Jung JY, Kang YA, Park MS, Kim YS (2019). Prognosis of small cell lung cancer with idiopathic pulmonary fibrosis: assessment according to GAP stage. J Oncol.

