# Supplementary figures and images for: Development and validation of a fourteen- innate immunity-related gene pairs signature for predicting prognosis head and neck squamous cell carcinoma
Source: BMC Cancer. 2020 Oct 20;20:1015. doi: 10.1186/s12885-020-07489-7 (PMC7574345; doi:10.1186/s12885-020-07489-7)

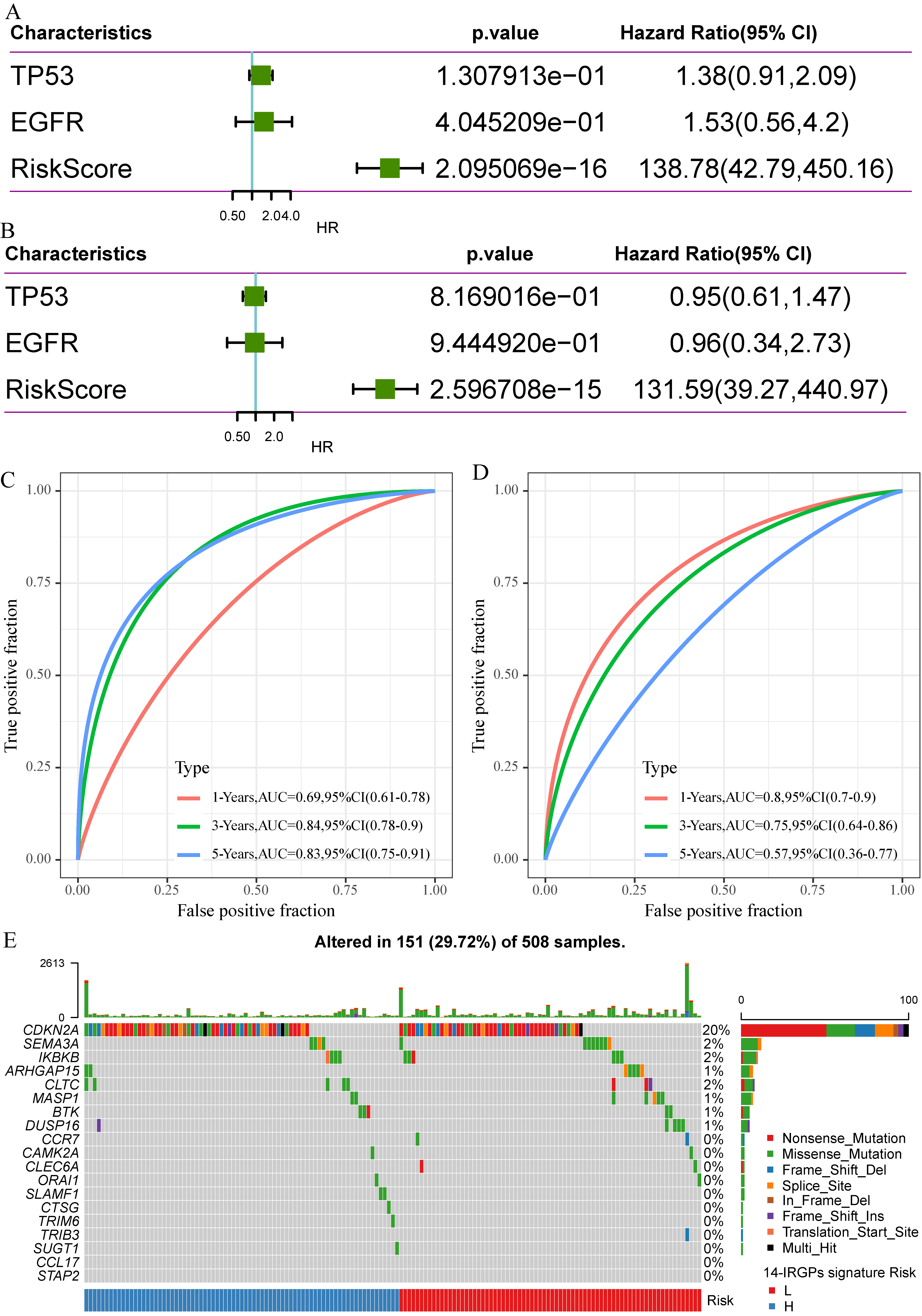

Supplement: Supplementary file 1 — Additional file 1. [file 12885_2020_7489_MOESM1_ESM.tif]
